# Hurler–Scheie syndrome in Niger: a case series

**DOI:** 10.1186/s13256-019-2047-2

**Published:** 2019-04-25

**Authors:** Hamid Assadeck, Moussa Toudou Daouda, Harouna Bako, Fatimata Hassane Djibo

**Affiliations:** 1 Department of Neurology, National Hospital of Niamey, PO Box 238, Niamey, Niger; 2 Department of Medicine and Medical Specialties, Faculty of Medicine and Pharmacy, Abdou Moumouni University, Niamey, Niger; 3 Department of Cardiology, National Hospital of Niamey, Niamey, Niger

**Keywords:** Mucopolysaccharidosis type I, Hurler–Scheie syndrome, Niger

## Abstract

**Background:**

Hurler–Scheie syndrome is an intermediate form of mucopolysaccharidosis type I which is a rare lysosomal storage disorder caused by the deficiency or complete absence of enzyme alpha-L-iduronidase activity. We report the first documented cases of Hurler–Scheie syndrome observed in Niger in a Touareg family.

**Case presentation:**

We studied the case of two 12-year-old twin Touareg boys and their 10-year-old Touareg sister whose parents are first-degree cousins, and there was no history of similar cases in their previous generations. The diagnosis of Hurler–Scheie syndrome was considered in these patients on the basis of clinical and radiological arguments, with the highlighting of a deficiency of enzyme alpha-L-iduronidase in serum and leukocytes. The twins had presented the first symptoms at the age of 24 months and the diagnosis of Hurler–Scheie syndrome was made at the age of 12 years. In their younger sister, the first symptoms were observed at the age of 3 years and the diagnosis was made at the age of 10 years. The three probands were born after a normal full-term pregnancy and a spontaneous vaginal delivery according to their parents. Their birth weight, height, and head circumference were within normal limits according to their parents. The three probands were brought in for consultation for stunted growth, joint stiffness with gait disorders, deformities of the thoracolumbar spine, recurrent otitis media, decreased hearing, increased abdominal volume, snoring during sleep, and facial dysmorphism.

**Conclusions:**

Even in countries with limited access to diagnostic means, a good knowledge of the clinical manifestations of the disease can help to guide the diagnosis of mucopolysaccharidosis type I.

## Background

Hurler–Scheie syndrome is an intermediate form of mucopolysaccharidosis type I (MPS I) which is a rare autosomal recessive lysosomal storage disorder caused by mutations in the alpha-L-iduronidase gene, responsible for a deficiency or complete absence of enzyme alpha-L-iduronidase activity [1, 2]. It results, therefore, in a progressive intracellular accumulation of non-metabolized glycosaminoglycans (dermatan-sulfate and heparan-sulfate), which is responsible for the multiorganic damage. The estimated prevalence of attenuated forms of MPS I (Hurler–Scheie and Scheie syndromes) is 1 in every 500,000 live births [3]. We report the first documented cases of Hurler–Scheie syndrome observed in Niger in a Touareg family.

## Case presentation

**Fig. 1:**
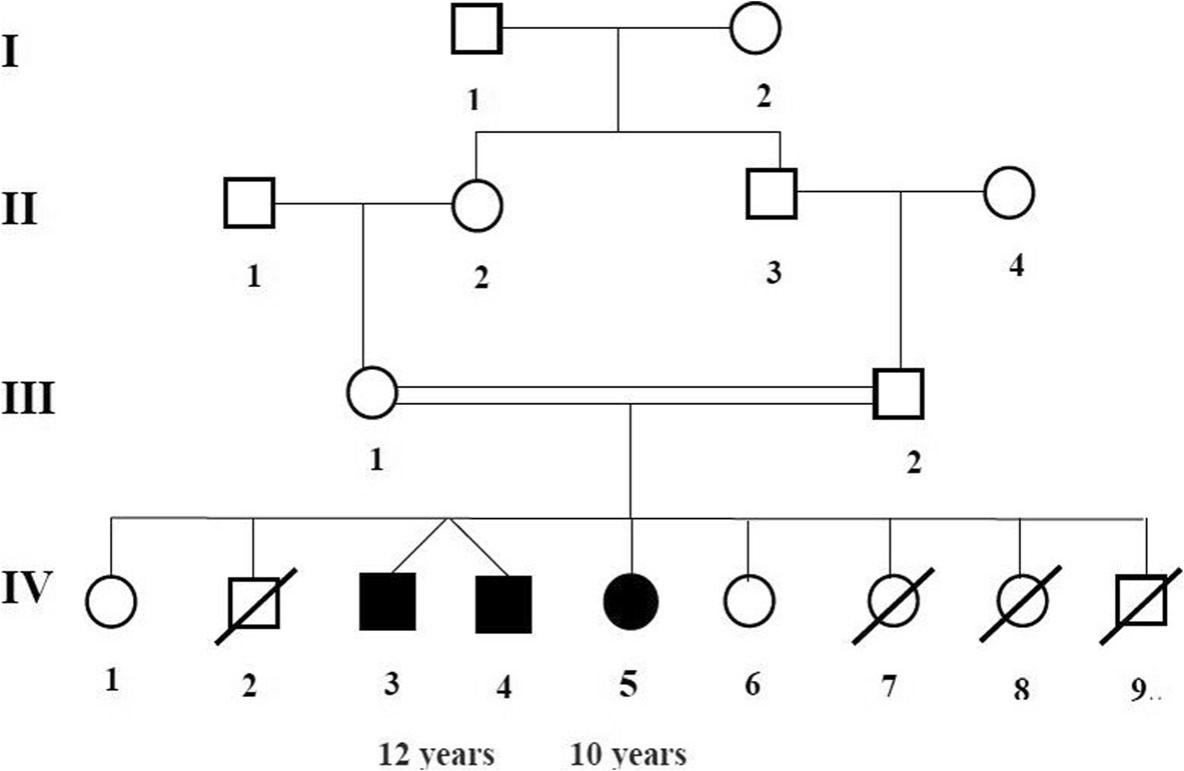
Family pedigree of the current patients

**Fig. 2:**
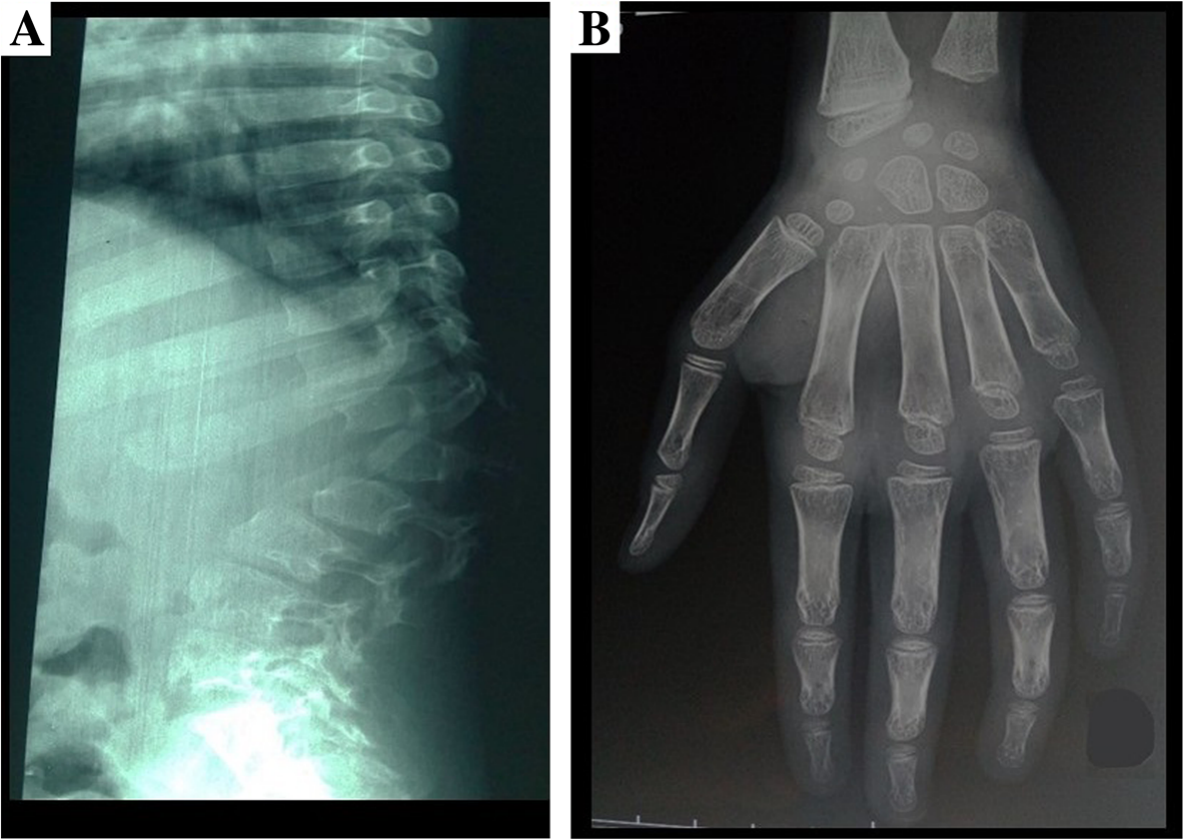
Skeletal X-rays showing anterosuperior hypoplasia of the vertebrae D12, L1, and L2 with kyphosis (**a**), and conical aspect of the distal ends of the phalanges (**b**)

The probands are two 12-year-old Touareg boys who are twins (patient IV/3 and patient IV/4, Fig. 1) and their 10-year-old Touareg sister (patient IV/5, Fig. 1). The three probands were born after a normal full-term pregnancy and a spontaneous vaginal delivery according to their parents who are first-degree cousins. Their birth weight, height, and head circumference were within normal limits according to their parents. The patients IV/3 and IV/4 were brought in for a consultation by their parents for stunted growth, joint stiffness with gait disorders, and deformities of the thoracolumbar spine. The first symptoms were increased abdominal volume, recurrent otitis media, umbilical hernia, and bronchial congestion with snoring during sleep, observed at the age of 24 months. Over time, the parents also noticed increased head volume, stunted growth, deformity of the spine, and decreased vision and hearing. Both patients also suffered from joint stiffness with a limitation of knee and shoulder movements and difficulty walking. They had a significant decrease in their walking perimeter due to joint stiffness and dyspnea of effort. Patient IV/3 had lost the ability to walk at the age of 11 years. Both patients did not have language disorders. They had slight intellectual disturbances. Their clinical examination found a short size of 103 cm, a facial dysmorphism with a short neck and micrognathia. They also had thoracolumbar kyphosis, and a prominent abdomen with hepatosplenomegaly and umbilical hernia. They had stiff and painful joints with limitation of active movements of abduction and antepulsion of the shoulders. Walking was impossible in patient IV/3 without human help. Patient IV/4 had paralysis of the right external oculomotor nerve. An audiogram found in both twins mixed bilateral deafness. An ophthalmological examination revealed a moderate decrease in visual acuity with corneal clouding in both twins. Cardiac auscultation revealed a mitral systolic murmur in patient IV/3. An electrocardiogram showed sinus tachycardia with signs of ventricular and atrial hypertrophy. Echocardiography showed mitral and aortic insufficiency in patient IV/3 and mitral insufficiency in patient IV/4. A chest X-ray showed cardiomegaly in both twins. An abdominopelvic ultrasound showed homogeneous splenomegaly and hepatomegaly in both twins. Skeletal X-rays showed anterosuperior hypoplasia of the vertebrae D12, L1, and L2 with kyphosis, and a conical aspect of the distal ends of the phalanges (Fig. 2). A cerebral computed tomography (CT) scan and brain magnetic resonance imaging (MRI) showed quadriventricular hydrocephalus with leukoaraiosis in both twins and an occipital arachnoid cyst in patient IV/4. Spinal cord MRI showed no particular abnormality. In the patient IV/5, the first symptoms were observed at the age of 3 years. She was brought in for a consultation at the age of 10 years at our request for a clinical evaluation. Her clinical history is similar to that of her older brothers but of less severity. Her clinical examination found a small size at 113 cm, a short neck, facial dysmorphism, prominent abdomen with hepatosplenomegaly, and umbilical hernia, without deformity of the spine. She did not have intellectual disturbances or language disorders. An ophthalmological examination found corneal clouding.

Considering the clinical history, clinical examination, and paraclinical examinations of our patients, a diagnosis of MPS I was suspected. Measurement of enzyme alpha-L-iduronidase activity (realized in France) highlighted a deep deficiency of enzyme alpha-L-iduronidase in serum and leukocytes. The search for mutations or deletions in the alpha-L-iduronidase gene has not been performed in our patients, as well as the measurement of urinary glycosaminoglycans. At the end of all examinations, the diagnosis of Hurler–Scheie syndrome was considered.

## Discussion

In this case series, we report the first documented cases of MPS observed in Niger in a Touareg family from the Agadez region, which is the northern part of Niger. The clinical history, radiological abnormalities of the skeleton, and deficiency of enzyme alpha-L-iduronidase allowed us to consider in our patients the diagnosis of the intermediate form of MPS I (Hurler–Scheie syndrome).

The attenuated forms of MPS I such as Hurler–Scheie and Scheie syndromes, are characterized clinically by later onset of symptoms, longer life expectancy, and mild or no central nervous system involvement. In Hurler–Scheie syndrome, the first symptoms of the disease usually appear after the age of 2 years with a median age of diagnosis from 4 years [4]. In our case series, the age at symptoms onset was 2 years in the twins (probands IV/3 and IV/4) and 3 years in their younger sister (proband IV/5) with the diagnosis age of 12 years in the twins and 10 years in proband IV/5. The clinical manifestations of Hurler–Scheie syndrome are diverse and include musculoskeletal manifestations (kyphosis, scoliosis, kyphoscoliosis, back pain, dysostosis multiplex, joint stiffness, valgus and varus deformities, and so on), respiratory and pulmonary manifestations (obstructive sleep apnea, asthma, snoring, recurrent bronchitis, and so on), coarse facial features, short neck, stunted growth of variable degree, macrocephaly, hepatosplenomegaly of variable degree, umbilical and inguinal hernias, ophthalmologic manifestations (glaucoma, optic atrophy, retinal degeneration, corneal clouding, blindness, and so on), cardiac manifestations (valvular disease, coronary artery disease, congestive heart failure, cardiomyopathy, myocardial infarction, and so on), otorhinolaryngological manifestations (chronic recurrent rhinitis, chronic recurrent otitis media, chronic sinus infections, hearing loss of variable degree, and so on), and neurological manifestations (myelopathy, hydrocephalus, carpal tunnel syndrome, and so on) [3–5]. In Hurler–Scheie syndrome, the intellect is normal or nearly normal [3], as in the case of our patients. The diagnosis of MPS I should be suspected in any person with the above-mentioned clinical manifestations [3]. In addition, coarse facial features and inguinal or umbilical hernia are early manifestations and the most prevalent symptoms in patients with Hurler and Hurler–Scheie phenotypes and should be considered early signs of a potential MPS I diagnosis [4]. Any suggestive clinical picture of MPS I must motivate the practitioner to practice the analysis of urinary glycosaminoglycans (heparan-sulfate and dermatan-sulfate) and the measurement of the lysosomal enzyme alpha-L-iduronidase activity. Thus, the diagnosis of MPS I is established in a proband with the suggestive clinical and excessive urinary excretion of glycosaminoglycans and either detection of deficient activity of the lysosomal enzyme alpha-L-iduronidase or identification of mutations or deletions in the alpha-L-iduronidase gene on molecular genetic testing [1, 3, 6, 7].

**Fig. 3:**
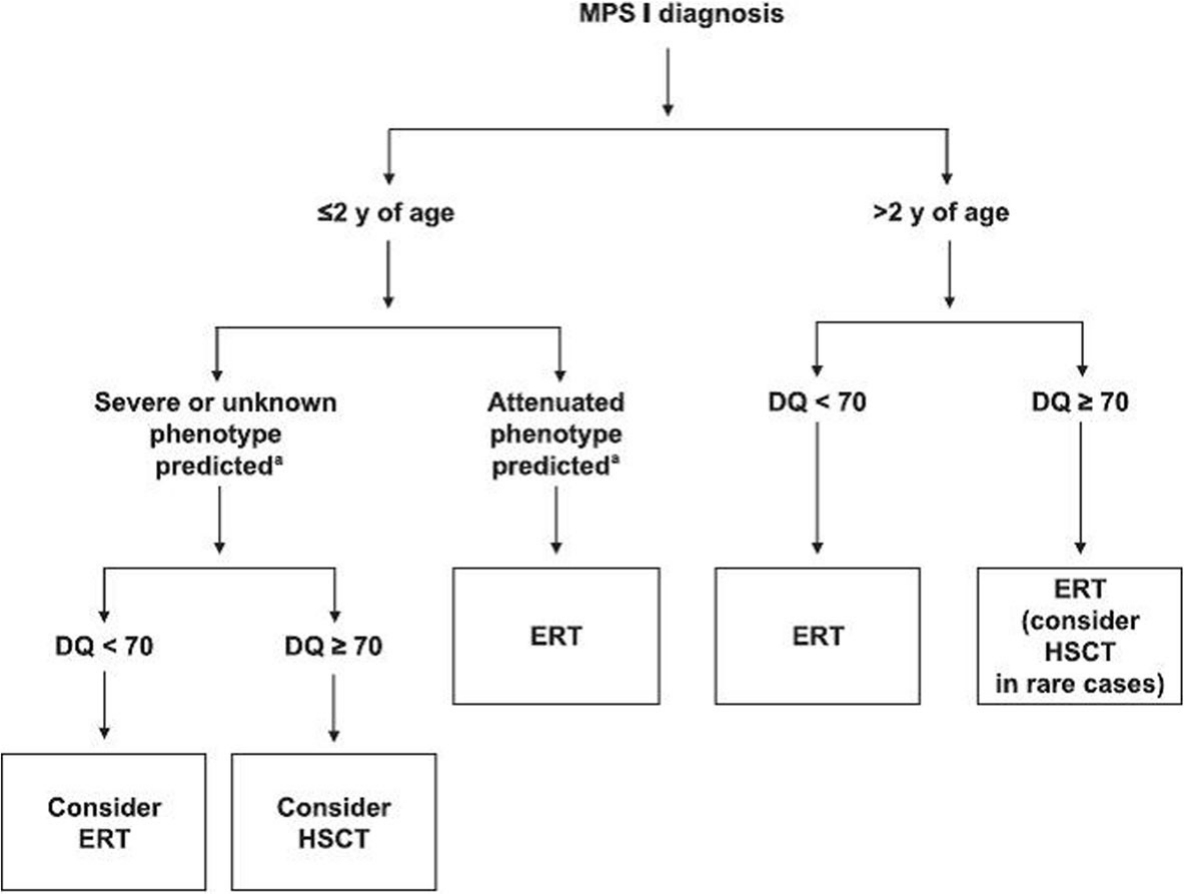
Treatment algorithm for patients with a diagnosis of mucopolysaccharidosis type I. *DQ* developmental quotient, *ERT* enzyme replacement therapy, *HSCT* hematopoietic stem cell transplantation, *MPS I* mucopolysaccharidosis type I, *y* year. (Fig. 1 from Muenzer *et al*., 2009 [7])

The management of patients with MPS I includes both preventive treatment and the treatment of severe manifestations [3, 7]. Preventive therapy includes enzyme replacement therapy (ERT) and hematopoietic stem cell transplantation (HSCT). These treatments allow patients affected with MPS I to obtain substantial clinical benefit for many disease manifestations, such as hepatosplenomegaly, upper airway obstruction, sleep apnea, cardiac symptoms, and coarse facial features [4–7]. The therapeutic choice must be determined individually for each patient with MPS I [7]. It should take into account the patient’s age, disease phenotype, developmental quotient (DQ), severity of clinical disease, and potential for growth. Figure 3 shows the treatment algorithm for patients with a diagnosis of MPS I. However, these treatments are effective only when they are initiated prior to the onset of irreversible organic lesions [4, 5]. In our case series, ERT would have been proposed in our patients, but, unfortunately, this treatment could not be realized because of the limited means of the parents of the patients and the unavailability of this treatment in Niger. The treatment of severe manifestations consists of the medico-surgical management of musculoskeletal manifestations (median nerve decompression, reparation of deformities of the spine, reparation of varus and valgus deformities, and so on), neurological manifestations (ventriculoperitoneal shunting for hydrocephalus with headache or loss of vision, spinal cord decompression in patients with cervical subluxation, and so on), respiratory and pulmonary manifestations (tonsillectomy for snoring or coarse breathing, continuous positive airway pressure for sleep apnea, and so on), cardiac manifestations (valve replacement, medical treatment of congestive heart failure, and so on), repair for hernias, and so on [3, 7–11].

## Conclusions

The clinical manifestations of MPS I usually appear in childhood in Hurler–Scheie syndrome, and their early recognition can lead to earlier diagnosis and early initiation of treatment, which may in turn lead to better patient outcomes.

## Abbreviations

CT: Computed tomography
DQ: Developmental quotient
ERT: Enzyme replacement therapy
HSCT: Hematopoietic stem cell transplantation
MPS I: Mucopolysaccharidosis type I
MRI: Magnetic resonance imaging
